# Hammer on

**Published:** 1876-11

**Authors:** 


### HAMMER ON.

FORTUNATE are they who, while they are living, can witness the fruit of their good doings, and are further rewarded by evidences of a grateful appreciation on the part of those for whose benefit they live and labor.

A worthy and conscientious clergyman—and there are many such—was greatly distressed in the inability to see that he was living to purpose: his advice was unheeded; his counsels disregarded, he seemed to himself like one who was beating the air, and that the work of his hands was labor lost. Nor was this the greater trouble; with the humility which belongs to a good heart, he began to lay all the blame on himself; to feel that he was not only not doing any good, but was hindering some other and more able laborer from saving the "lost." In such a frame of mind, scarcely knowing what to do, it is related that he had a dream, to the effect, that with the tiniest hammer, he was appointed to break in pieces an immense rock. The task seemed utterly hopeless, yet he did as he was bid, without seeing that he made the slightest impression, and he was on the very point of exclaiming, in irrepressible impatience, "Wherefore all this useless labor?" when the huge mass crumbled to pieces. He was one of that happy class of minds which endeavored habitually to see the hand of Providence in all that occurred, and felt and sang

"In each event of life how clear,
Thy ruling hand I see."

And with unwonted alacrity, he girded himself to new and wider labors, with the result of gathering such a harvest as in his wildest imagination he never dreamed of.

Laudanum, paregoric, [illegible]orphine, for no mixture ever sold for coughs, colds, and [illegible]umption, ever, by any chance, fails of one of these ingre[illegible] Such mixtures always soothe, always diminish the cough, causing a belief that they are doing good, hence they are more freely and frequently taken. It is precisely as if, when the hold of a ship is on fire, the hatches are closed, and because no more smoke is seen to arise, the captain should immediately place himself at ease, under the conviction that the fire is out, while in reality it is but kept under, gathering force, only a little later to break forth with resistless power.

Opium does not cure any thing; it never did. All that can be scientifically claimed for it is, that it gives time to nature or the physician, as does closing the hatches when the hold is on fire; but if the time is not wisely improved, disaster must follow.

Cough, in a cold, is nature's effort to eject from the lungs what does not properly belong there; precisely as when a crumb "goes the wrong way," that is, into the windpipe instead of the stomach. In each case opium diminishes the sensibility of the parts so that they do not feel the presence of the offending particles, and nature is cheated. Any one can see that because a dose of morphine quiets the cough from a crumb in the windpipe, it does not "cure," it does not bring it away. Most precisely so is it with phlegm in the lungs; it has the effect to keep it there to accumulate, just as water does in the spout of a pump, when little boys close the mouth of it with their hands, pumping on all the time. Here the comparison ends. However long the water is kept in, it is water still. Not so with phlegm in the lungs; every instant it is kept there, the heat of the parts being a hundred degrees of Fahrenheit, it evaporates, becomes less watery, and of consequence, more tough, harder to dislodge, increasing in bulk by accumulations which also grow thicker by evaporation, until "the pipes" are plugged up with tough phlegm so that the air passes it with increasing difficulty, and the patient wheezes like—an asthmatic; the lungs labor fearfully for breath, they can not get enough air, hence the blood becomes impure and thick, and in this condition goes to the heart, which labors to send it on through the body, but can not wholly empty itself at each beat, as is natural; and the blood still pouring into it from the lungs, it gets preternaturally full, hence its fibers are relaxed and then distended: this is "enlargement of the heart." For a while nature struggles to relieve herself of this surplus, until the laboring heart has expended all its strength in the vain effort, until it can not give another beat, and suffocation is the instantaneous result.

Many a man before now, has been literally "killed with kindness," as it would seem was the fate of Washington Irving, of whom it may be truthfully said, he was kindness personified. No sooner does a man show that he has a cold, than advice is protruded in the glibbest manner possible by every second friend he meets. Let the reader learn by the sad narration given, never to give or take advice, even in so simple a thing as a common cold. Let counsel come in all cases from common-sense or a physician.

